# Assessing the Sociocultural Impacts of Emerging Molecular Technologies for the Early Diagnosis of Alzheimer's Disease

**DOI:** 10.4061/2011/184298

**Published:** 2011-09-21

**Authors:** Marianne Boenink, Yvonne Cuijpers, Anna Laura van der Laan, Harro van Lente, Ellen Moors

**Affiliations:** ^1^Department of Philosophy, Faculty of Behavioral Sciences, University of Twente, P.O. Box 217, 7500 AE Enschede, The Netherlands; ^2^Department of Innovation Studies, University of Utrecht, 3584 CS Utrecht, The Netherlands

## Abstract

Novel technologies for early diagnosis of Alzheimer's disease (AD) will impact the way society views and deals with AD and ageing. However, such “sociocultural” impacts are hardly acknowledged in standard approaches of technology assessment. In this paper, we outline three steps to assess such broader impacts. First, conceptual analysis of the ideas underlying technological developments shows how these technologies redraw the boundary between Alzheimer's disease and normal ageing and between biological and social approaches of ageing. Second, imaginative scenarios are designed depicting different possible futures of AD diagnosis and societal ways to deal with ageing and the aged. Third, such scenarios enable deliberation on the sociocultural impact of AD diagnostic technologies among a broad set of stakeholders. An early, broad, and democratic assessment of innovations in diagnostics of AD is a valuable addition to established forms of technology assessment.

## 1. Introduction

The early detection of Alzheimer's disease is a widely shared goal in current biomedical research. At many labs and in many hospitals around the world, scientists are working hard to develop knowledge and technologies to enable such early detection. Most attempts are focused on the identification of biomarkers that might indicate early stage Alzheimer's or even predict the disease. Recent recommendations and proposals to adapt the definition of and the guidelines for diagnosing Alzheimer's disease reflect these endeavors. The new guidelines by the American National Institute on Aging and the Alzheimer's Association conceive AD as a three-staged disease process, including a preclinical stage, a stage of mild cognitive impairment, and the final stage of Alzheimer's dementia. The authors of these guidelines suggest that biomarkers can play a role in delineating the preclinical as well as the mild cognitive impairment stage and might be used to increase the certainty that AD pathophysiology is the basis of the clinical syndrome [1, 2]. In the proposals for the DSM V, Alzheimer's Disease is included as a subtype of major as well as minor neurocognitive disorders. In the case of minor neurocognitive disorders, the category most relevant to early detection, the proposal requires not only evidence of memory complaints, but also “supporting evidence for the Alzheimer etiology (e.g., a positive test for a known mutation in an Alzheimer's disease associated gene) or with evolving research, documentation based on biomarkers or imaging” [3]. In both the NIA/AA guidelines and the DMS V proposals, then, molecular biomarkers are expected to play an important role in delineating and diagnosing stages preceding full-blown AD, even though their precise contribution is still unclear.

This does not mean, however, that early detection of Alzheimer's Disease is uncontroversial. Researchers working on such early detection may be confronted with questions about, for example, the value of early detection when a cure for Alzheimer's is still lacking, about the potential medicalization of the ageing process, about the right of potential Alzheimer patients/families to codecide on a technology that might have a great impact on their lives, and about the distribution of liabilities. These are difficult questions to deal with, and scientists may not be in the best position to answer them. From a societal perspective, however, it is important that such considerations are taken into account before early detection for Alzheimer's disease is introduced at a wide scale. 

Health technology assessment (or medical technology assessment) is often used by policy makers to judge the desirability of novel technologies before they are introduced. However, most forms of HTA are limited in scope. They focus on the clinical efficacy of the novel diagnostic or therapeutic tool, its potential risks or side effects, and its efficiency. The fact that those technologies might also have an impact on the organization of medical practice and on society and culture at large, is neglected. Such sociocultural impacts are, of course, difficult to foresee, let alone to quantify. This does not make them less relevant, however. If we want to include such broader impacts, a different type of assessment is needed in addition to the quantitative tools of HTA.

In this paper, we describe how sociocultural impacts (in particular impacts on views on and practices of ageing) could be included in the assessment of emerging diagnostic technologies for Alzheimer's disease. After explaining the practical and theoretical background of the research from which this paper resulted, we will outline the three steps of our approach. First, a conceptual analysis of the ideas underlying technological developments can show how a new type of diagnosis might redraw the boundary between Alzheimer's disease and normal ageing. Second, we investigate how possible futures can be represented to stakeholders. A promising route is to produce imaginative sociotechnical scenarios depicting different possible future ways to deal with AD, ageing, and the aged. Third, we review the possibilities for deliberation on the potential sociocultural impacts. Scenarios like the ones outlined here can be presented to a broad set of stakeholders (including patients, families, medical professionals, medical industry, policy, and insurance companies), which allows an interactive assessment of sociocultural impacts. We conclude that the potential of current innovations in diagnostics of AD merits a democratic assessment, including an early and broad assessment of sociocultural impacts.

## 2. Practical and Theoretical Background

The work described here originated in the context of a multidisciplinary research project, in which philosophers, ethicists, and social scientists cooperate with a Dutch biomedical research consortium developing molecular tools for early detection of Alzheimer's. The biomedical research project is called the “Leiden Alzheimer Research Nederland” or LeARN-project. Partners in the consortium are Leiden University Medical Centre, VU University Medical Centre, University Medical Centre St. Radboud, Maastricht University Medical Centre, Philips Electronics, Schering Plough (part of MSD), BAC, Cyclotron and Virtual Proteins. The LeARN-project is funded by the Centre for Translational Molecular Medicine. (For more information on the CTMM and the LeARN-project, http://www.ctmm.nl/.) The multidisciplinary project from which this paper originates uses the LeARN-project as a case study, but is independently funded by the Dutch Organization for Scientific Research (NWO). The LeARN consortium aims to develop tools for early diagnosis of AD. Three types of medical technology are used to achieve this goal: PET scans, MRI scans, and CSF analysis. The biomedical project investigates which (combination of) molecular biomarkers are able to detect AD in a sufficiently reliable way. The aim of our multidisciplinary project is to contribute to responsible innovation by anticipating the potential social and ethical aspects of early (molecular) diagnosis of AD as developed within the LeARN project. Our reflections on the potential impact of this type of emerging technologies, however, are relevant to any attempt to find molecular biomarkers for early diagnosis of AD. 

One of the starting points of our project is the observation that new biomedical technologies may have a broad set of impacts on medical practice, society, and culture, but that society hardly takes these into account when assessing the desirability of a novel technology. This is unfortunate, both from an ethical as well as a practical point of view. In an ethical sense, responsible innovation means that the innovation at hand should be acceptable to all those for whom something is at stake in the innovation process. And this, in turn, presupposes that these stakeholders have the opportunity to reflect on all types of considerations on the desirability of an innovation [4, 5]. A more democratic innovation process should address this wider set of considerations. On a practical note, anticipating the potential broader impact of a technology helps to prevent unpleasant surprises, or even a backlash, later on [6–8]. As, for example, scientists working on the genetic modification of crops learnt to their dismay, not all innovation that seems useful to science is embraced by a lay public. Many medical innovations as well never make it to daily use in health care because they do not fit the needs and values of their targeted users. Anticipating the impact of emerging technologies in society may contribute to more robust and useful technologies.

This is, however, hard to realize. After all, emerging technologies do not exist yet and are characterized by many uncertainties. It is not clear yet what they will look like, how they will perform, and how they will be used, and this makes it difficult to determine how desirable they might be for future users. It may even be difficult to identify who these users will be. In the case of early detection for AD, for example, it is far from clear which (set of) molecular biomarkers will prove to be sufficiently reliable (if any), how useful they will be for prognosis, who would use this type of diagnostics, and in what way. This might be an argument to first wait and see and not to speculate on things to come before the technology has evolved to the stage of a prototype or can be experimented with. However, in social studies of science and technologies, it is well known that at such a later stage it is much more difficult to steer technology development in a different direction. So, we may be caught in what Collingridge [9] called the “dilemma of control”: at an early stage of technology development, it is difficult to foresee what the technology will be like and what it will do, but at a later stage it is too late to effectively steer the development.

One approach developed in science and technology studies to circumvent this problem is “constructive technology assessment” (CTA) [6, 10]. Starting from the assumption that early anticipation of future developments is necessary to enable any attempt at socially orienting technology development, this approach aims to engage stakeholders early on. In such engagement activities, ideals and expectations guiding the work of technology developers can be scrutinized and opened up for broader deliberation, ultimately leading to a broadening of the criteria used to design a socially and ethically desirable technology. 

One of the tools used in engagement activities are “sociotechnical scenarios” that tease out the different ways in which technology, society, and culture (including morality) might interact with each other [11, 12]. The use of scenarios has its origin in business strategy and later on also in policy studies [13]. In particular, the famous Shell group developed a methodology to produce and use scenarios [14, 15]. Scenarios are neither predictive, nor completely fictitious. In general, they are used to test whether strategies are robust under unexpected future circumstances. The plural character of scenarios enables business leaders or policy makers to prepare for the future, whatever in the end will be realized. Since they are grounded in both historical analysis and an exploration of how different stakeholders respond to the developments at hand, the narratives can be characterized as products of “controlled speculation” [16]. Using such scenarios in focus groups or interactive workshops with stakeholders enables sensible deliberation on emerging technologies, while at the same time acknowledging the uncertainties involved [10].

The project described here also attempts to create space for stakeholder engagement early in the process of technology development and to provide input for such engagement in the form of controlled speculation on the future. More than other CTA projects, however, it builds on a conceptual analysis of the mutual interaction between concepts of disease (i.e., AD) and ageing on the one hand, and technology development on the other. As argued by Boenink [17], the analysis of emerging shifts in the concepts of disease and health helps to anticipate ethical issues related to emerging biomedical technologies. Whereas the particular direction and performance of an emerging technology may still be uncertain, the underlying framings of disease and health, or normality and abnormality, are often visible at an early stage already. Such conceptual shifts help us to anticipate subsequent shifts in the organization of health care and nonmedical practices, thus opening them up for ethical and social debate. This is particularly true for technologies related to Alzheimer 's disease, since the history of Alzheimer's disease shows how novel diagnostic and therapeutic technologies for AD framed the disease and, by implication, also views of ageing in new and unexpected ways [18, 19]. Moreover, as Joyce and Loe [20] argue, technology in general is an important element in the imagery, expression, and performance of modern ageing. The first step of our approach, therefore, aims to reconstruct the history of Alzheimer's disease as a concept more or less distinct from other diseases as well as “normal ageing,” and to investigate how the existing boundaries between AD and ageing might be redrawn by research into the biology of AD.

## 3. Technology, Alzheimer's Disease, and Ageing

Several authors have reconstructed and analyzed the history of Alzheimer' disease [18, 19, 21]. They all share the observation that the label “Alzheimer's disease” has been interpreted and used in various ways since Kraepelin included it in his nosology of psychiatric disease in 1910. Both scientific and technological developments and developments in society have influenced the way the disease was framed. The evolving definitions, in turn, have significantly determined how society deals with elderly people displaying complaints and signs that tend to be associated with dementia. Definitions of Alzheimer's disease have coevolved with scientific and technological as well as social developments. We will highlight here a few excerpts of this history of interacting technological and sociocultural developments, as a starting point to explore how it might further evolve in the future. 

The case Alzheimer himself described in 1907, which would later become the basis for Kraepelin's definition of AD, was about a 51-year-old woman, Auguste D., who displayed clinical features of what was then called “senile dementia.” What initially distinguished her case from other cases of senile dementia was her relatively young age. At autopsy, however, Alzheimer observed another difference. The brain tissue displayed not only the plaques often associated with senile dementia. By using a newly developed silver staining technique, Alzheimer also made visible specific neurofibrillary tangles [22]. It soon became clear that these tangles were present in many other cases of senile dementia as well, including those of elderly patients. When Kraepelin defined “Alzheimer's disease” a few years later, he therefore focused on the *clinical* criterion of age; according to his definition, AD was a form of *pre*-senile dementia. This immediately engendered a discussion on the question whether the age difference is sufficient to identify AD as a disease sui generis (Alzheimer himself was among those who thought it was not) and how both presenile and senile dementia relate to ageing processes in general [18]. The identification of AD as distinct from senile dementia seemed to suggest that, whereas the AD was clearly distinct from the normal ageing process, senile dementia might not be so different from normal ageing after all [23]. 

Notwithstanding such debates, the age-based criterion continued to be used for diagnosing AD till the 1970s. As a result, the prevalence of the disease was not very high. Only after the age criterion was dropped in the 1970s, the number of people diagnosed with AD rose steeply [18]. This evolution from “presenile” and “senile dementia” to “dementia (including the Alzheimer type)” can be understood as the result of converging social and technological developments that had been going on in the 1960s. On the one hand, the position of elderly people in society had changed. With the growing number of individuals living well beyond 65, the “third age” started to be regarded as an attractive period of life. Ageing need not to be feared, and if it was accompanied by marginalization and stigma, this was due to social processes that should be changed. The concept of “ageism” was coined in 1968 by gerontologist Robert Butler to denounce all attempts to stereotype and discriminate people just because they are old. From this perspective, labeling elderly people as “senile” often was an easy way not to take them seriously. It became politically relevant to distinguish the broad, pejorative use of the term senility from a careful biomedical diagnosis of dementia. Such a diagnosis would at the same time help to protect the golden shine of the third age against the gloomy shadow of deterioration during the fourth age. 

On the other hand, several developments in science and technology intensified the search for a correlation between the clinical symptoms of senility and pathological signs in the brain [24]. The invention of novel counting methods for the number of plaques and tangles in brain tissue, and the emergence of electron microscopy, enabled novel takes on the pathology related to AD and senile dementia. The counting methods were thought to show that the density of plaques relates to the severity of the clinical phenomena and thus to make a case for a clear biological substrate for these symptoms. Electron microscopic investigations of plaques and tangles revealed that these had different morphological features. The plaques were identified in 1964 as consisting of amyloid; the substance of the tangles would remain obscure for some more time. The findings raised all kinds of novel hypotheses with regard to the biological process underlying the formation of plaques and tangles and their role in causing the clinical symptoms. Although the causal process itself was not clear at all, the idea gained ground that dementia and Alzheimer's disease were essentially biological processes and should be diagnosed and treated on that level [23]. As a result, the relative importance of clinical symptoms and biological signs was reversed. The presence of brain pathology became a prerequisite for diagnosing dementia in general (including Alzheimer's) [18].

Another significant shift in the framing of Alzheimer's disease and ageing occurred in the 1970s and 1980s, when neurotransmitters became an important focus of interest, resulting in a strong focus on memory complaints. The interest in neurotransmitters was due to developments in therapeutic technology, which started with the observation that drugs blocking cholinergic activity in the brain (prescribed to women in child birth) produce dementia like memory disruptions. This was further developed into the hypothesis that a lack of acetylcholine in the brain led to neuronal death and subsequently to problems with short term memory—the so-called “cholinergic hypothesis.” This hypothesis led to several attempts to develop drugs countering this cholinergic deficit, resulting in cholinesterase inhibitors like tacrine. The effects of these drugs were limited, because they only slow down the degradation of acetylcholine. When a substantial number of neurons have died, the effect of the drugs will decrease. Accordingly, cholinesterase inhibitors can only be effective in the early stages of dementia.

What is striking in this episode from the history of Alzheimer's disease is that the complex phenomenology of the disease was narrowed down to memory loss. Whereas the definition of AD from the start had included a broad set of symptoms, including other cognitive problems and personality problems, scientific and clinical attention now focused on the loss of memory functions. Since deteriorating memory is associated with ageing anyway, this narrow focus invited a reconnection of AD and ageing. This connection is still visible today, in the often heard half cunning, half anxious comment that “this shows I'm off on the Alzheimer track” when a (in all likelihood minor) slip of memory has occurred. The promises of drugs slowing down the process of memory loss thus lead to an increased social anxiety about forgetfulness, and a concurrent neglect of the other symptoms earlier is thought to be correlated with AD and dementia.

The most recent episode highlighted in the history of AD is the flurry of genetic and subsequent molecular research that started in the 1990s. With the invention of transgenic mouse models and the rise of genetic research in humans, the hunt was open for “the” gene causing AD. As with most other diseases, results were more modest than hoped for. To date, four genes have been identified that are related to AD. Three of them (called APP, presenilin-1, and presenilin-2) are linked to cases of AD beginning at an early age, and they are usually transmitted in an autosomal dominant way. These monogenetic variants of AD account for less than 1% of all AD cases. A fourth gene (APOE) is associated with an increased risk of AD in the general population (usually also associated with early onset of the disease) and is thus one cause among others in a multifactorial variant of AD [25]. Here, as before, the novel technologies engendered a shift in the conceptualization of AD, now producing a distinction between genetic (or familial) and sporadic AD, which seemed to be associated with, but not identical to, a distinction between early and late onset AD. The identification of specific genetic risk groups has driven attempts to actively screen individuals from such families with offers of genetic testing; however, most countries do not offer population screening for the common late-onset form of AD [26].

Even though the genetic variants of AD do not seem to be very prevalent, molecular research into the function of the APP gene (and later also into the presenilin genes) led to an influential hypothesis about the causal pathway underlying AD: the amyloid cascade. The idea that AD is a disease with progressive stages had been around for decennia [23]. The amyloid cascade hypothesis gave it a clear pathological foundation, by tracing the presence of amyloid plaques and the subsequent neuronal death back to genetic alterations causing excessive production of A*β*42 protein [27, 28]. The hypothesis, although not without critics and having evolved substantially since it was first proposed in 1991, is quite influential in current AD research. It has strongly reinforced thinking of AD as a gradual process, in which biological changes precede clinical manifestations. This helped to make sense of the long standing finding that some individuals who during life did not display any clinical symptoms, at autopsy do show the plaques and tangles characteristic for AD. The conceptualisation of “a-symptomatic AD” in turn stimulated proposals to develop molecular tools for early diagnosis of AD.

Despite the recent emphasis on the biological processes of AD, several authors stressed the importance of clinical manifestations as well. Petersen et al. (1999) showed that there is a group of people experiencing cognitive decline, who do not satisfy current diagnostic criteria for AD. Analogous to the presupposition of a gradually developing process on the molecular level, they proposed to use the label of “Mild Cognitive Impairment” for this group and suggested that it may be a transitional stage between normal functioning and dementia in general or AD in particular [29]. The value of this concept is currently highly debated, as it is unclear how it might relate to biological processes [30]. It is clear, however, that current thinking on AD both on the molecular and the clinical level tends towards conceptualising the disease in terms of a gradual process with signs and symptoms that become more manifest as time progresses.

## 4. The Future of AD and Aging: Some Scenarios

Given these ongoing changes in the clinical practice and understanding of AD, the question is how the envisioned early diagnosis will affect the social and cultural meaning of the disease and of aging in general. To assess such broader impacts, we developed “possible futures” or scenarios serving two goals. First, they invite reflection on the broad range of impacts technological development in the field of AD diagnostics may have. In addition, they enable early and public deliberation on the desirability of such impacts, as well as identification of the conditions for creating a desirable future. When subjected to debate among a broad set of stakeholders, such scenarios can help to democratise technological development and steer it in a socially and ethically desirable direction, thus contributing to a responsible innovation process. 

The scenarios presented here share a general starting point: they presuppose that attempts to develop biomedical technologies for early detection of AD, in one form or another, will not suddenly disappear. Their form and societal impact will depend, however, on the actual path of development and their success, as well as their embedding, all of which are unknown right now. These uncertainties can be reduced to some extent, however, by three observations based on the history of AD and ageing presented above. First, promises and expectations with regard to explaining and curing AD, even when widely shared in the research community, often do not materialize. Second, up to now, it has been difficult to explain AD in terms of a single, linear causal pathway. And finally, opening up bodily processes that were formerly invisible often produces a fragmentation of what counts as “normal bodily functioning.”

Starting from these general observations and inspired by the specific conceptual and historical developments sketched in Section 2, we developed three scenarios. In the first, we explore what might happen if current attempts to identify molecular biomarkers for AD are successful. The second scenario, in contrast, outlines a future in which it proves to be difficult to associate AD unequivocally with underlying biological processes. The third scenario depicts a future (further away in time than the first two) in which initial failures to identify informative biomarkers lead to novel technological developments. 

### 4.1. Scenario 1: Forgetfulness as a Biological Problem

This scenario starts from the assumption that the promise of molecular research is indeed realized: (a set of) biomarkers is/are identified that neatly distinguishes a specific group of people from others, in terms of their chance to develop clinically manifest AD. Not only a substantial part of those patients diagnosed with AD score positive on the biomarker, but also many of those with MCI. This greatly reinforces the legitimacy of the MCI label, which until recently was still contested as a pseudodisease. In due time, research shows that those MCI patients scoring positive on the biomarker test are significantly more likely to develop full-blown AD in the years to come. This motivates several memory clinics to experimentally introduce the biomarker test in their diagnostic workup. There is some professional and societal debate, however, whether it is ethically sound to offer this test in the absence of a cure for AD. What use is it to know that you are off on a prospect of gradual decline if nothing can be done about it? Some neurologists argue that the test may offer reassurance to those who test negative. Psychiatrists and gerontologists point out that although a cure is lacking, therapeutic options and different care arrangements are available. Knowing your biomarker status may help to make informed decisions with regard to the future. In The Netherlands, some voices claim that a positive biomarker test might enable people to lay down clear advanced directives at what stage they consider their life to have become worthless. This might help to solve the difficult situation AD patients applying for legal euthanasia. Currently, such requests are usually rejected, either because the patient's situation is not yet clearly hopeless, or because his/her mental capacities have deteriorated to such an extent that the request is not considered autonomous anymore. Others respond, however, that this is exactly the reason to abstain from biomarker testing. In the end, the novel professional guidelines for AD diagnostics advise to perform a biomarker test, but only when the patient is well informed about the implications, and when post test counselling is provided in case of a positive test result. 

As memory problems become clearly linked with biological functioning, subjective experience of one's cognitive functioning is more and more distrusted. Physicians are confronted with an increase of middle aged individuals wondering whether their cognitive functioning is deteriorating. Such worries are reinforced by self-tests for regular cognitive checkups available on the internet, aiming to sell training programs which promise to improve cognitive functioning. Some employers are quick to offer cognitive check-ups as a service to their employees, stating that testing is not meant to demote anybody, but might help to adjust the working environment to one's evolving capabilities. In general, however, there is a poor relation between the results of such tests and the biomarker test. Physicians, therefore, denounce these tests and advise to rely on medical diagnosis only.

In due time, the drugs with a modest effect only on AD patients are found to effectively prevent memory complaints in asymptomatic individuals who score positive on the biomarker test. This reinforces the demand for testing. It also leads to a medicalization of memory loss and an increase in social norms for cognitive functioning. Those who can no longer live up to these demands are even slightly blamed, because they could have opted for biomarker testing and drug treatment earlier. The government and employers start campaigns promoting “healthy ageing,” in which forgetfulness is presented as one of those annoying phenomena from the past. Ultimately, almost everyone above age fifty, only a few stubborn people excepted, starts using the biomarker test as a regular checkup. The test now actually serves as the new golden standard for diagnosing AD. Neuropsychological tests for diagnosing AD consequently almost go out of use. 

Only after a substantial number of years, psychiatrists observe that the ratio between patients with dementia and those with other diseases in mental hospitals and nursing homes has changed. The huge number of AD patients has decreased because people seek help earlier and memory complaints can be treated quite effectively. The number of patients displaying behavioural and personality disorders has increased, however, partly because these phenomena are now no longer associated with AD. The success of the biomarker tests, moreover, has decreased the funding as well as the attention for these other complaints, resulting in a nursing home population that is difficult to deal with. Physicians and nurses willing to work at such institutions are scarce; in particular the young ones shy away from a confrontation with a part of humankind that so obviously does not satisfy what one would expect humans to be.

### 4.2. Scenario 2: Biomarkers as an Add-On to the Diagnostic Toolbox

Despite the amount of time, work, and money spent on the identification of biomarkers for AD, the high expectations of AD research at the beginning of the 21st century do not materialize. MRI techniques seem to be able to single out some groups of patients highly likely to develop full-blown AD, but this targets only a very small subset of the whole AD population. Moreover, quite a few patients are excluded from having a 7T MRI because they have a pacemaker or other metal implants. Insurance companies are not willing to pay for MRI scans with an apparently low added value, thus in fact blocking the inclusion of this technology in the diagnostic workup. Biomarkers identifiable in CSF do slightly better: a CSF test combining several biomarkers does identify a large subset of MCI patients likely to develop AD. However, whereas it predicts quite well the onset of memory complaints, there is no clear correlation with other symptoms of AD. At consensus meetings to prepare novel professional guidelines for AD diagnostics, there is huge controversy whether or not to include the CSF test. Ultimately, most countries include the test in the guidelines, but only in conjunction with other laboratory and clinical tests aiming to identify the full spectrum of potential AD symptoms. 

With the increase of potential tests included in the diagnostic workup, however, the chances of test results contradicting each other increase as well, complicating the diagnostic process. Some medical professionals start talking in terms of “proven” and “suspected” cases of AD—with proven referring to the presence of amyloid that is made visible in vivo for the first time in those that test positive on the CSF test. Others argue that it would be more precise to distinguish “amyloid deposit disease” (ADD) as distinct from AD and MCI. Pharmaceutical companies that have invested a lot of money in research how to counteract the amyloid cascade organize clinical symposia focusing at the novel ADD phenomenon, but they cannot hide that the results of their research efforts are as yet very limited. 

On a less grand scale, research into the role of lifestyle and environmental factors in causing AD continues. The lack of clear categories and associated therapies does engender a lot of confusion and anxiety among elderly. Patient organizations for AD start campaigns to promote regular cognitive check-ups for elderly (using the slogan “Don't forget to check your memory!”) and make such cognitive self tests available on their website. If the result is below average, people are advised to see a physician and ask for AD diagnostics including the CSF test. This medical orientation is contested by some physicians, elderly, and family members of AD patients, leading to a split in the patient organization. The mission of the novel patient organization is to help people live well with dementia. This organization supports projects designing new forms of housing or implementing new ways of working with elderly in organizations, in due time claiming some successes in improving quality of life for both patients and their social environment. Most elderly experiencing cognitive or behavioural problems, however, are disappointed if they do not test positive on the CSF test but receive the label MCI or “suspected” AD instead. They feel they are sentenced to a vague but deadly disease, without any prospect of a future cure. Moreover, both their informal caregivers and the biomedical professionals start treating them as if they were completely helpless already. “It's really annoying,” a lady from the patient organization says. “Most of my friends have given a key of their house to the neighbours, in case they might forget their key. But if I stand some time in front of my door to find my key, my neighbour comes out and says: Did you forget your key again? Let me open the door for you!”

### 4.3. Scenario 3: The Normal Becomes Personal

Ongoing AD biomarker research definitely produces a huge increase in data on individual bodily functioning, but it is difficult to identify biomarkers that are sufficiently informative about chances of developing AD. One of the problems is that many biomarkers characterise very small groups only. Moreover, those biomarkers that do identify substantial groups at risk of AD often lead to conflicting results, which makes them difficult to interpret. This lack of clear progress makes funding organizations wary to finance more biomarker research, and for some time developments in this domain come to a halt. Developments in the technology platforms enabling biomarker tests do continue, however. 

An important step is made when blood serum proves to be as reliable as source of biomarkers as CSF. In addition, lab-on-a-chip technology or even ingested sensors that communicate measuring results to a computer system become available, making it possible to regularly perform biomarker tests in a way that is hardly burdensome to the test subjects. This motivates some researchers to start monitoring a set of biomarkers in healthy individuals and MCI and AD patients. When this ambitious and time-consuming study is completed, huge variations within these groups (instead of between them) come to the fore. Subsequent prospective research, with a larger set of healthy individuals, points out that what is normal for one may be quite abnormal for another person. On the long term, however, deviations from one's personal pattern prove to be informative with regard to a person's chances to develop complaints related to AD. 

After many years, then, the procedure for diagnosing AD is radically transformed. Instead of having a one-time set of tests in the memory clinic, people are sent home with a monitoring device. What is more, physicians and patient organizations alike start urging middle-aged people to have the personal pattern of their AD biomarkers established before decline sets in, because only this will provide a good reference point for later diagnosis. The roles and responsibilities of clinicians and patients are radically redistributed, with the latter ones taking an active part in diagnosis. Some patients (or rather clients, since they are healthy individuals) become quite good at interpreting their test results in view of their own functioning, deciding for themselves which deviations are significant and which ones are not. When changes are deemed significant, a set of therapeutic options is advised. Next to the prescription of drugs (which still have a limited effectiveness), clients are urged to continue working and be productive as long as possible, exercise regularly, and eat healthy.

 In general, people become more aware of their own ageing process. Since they are regularly confronted with changes in bodily parameters, they become also more keen on identifying changes in their physical, cognitive, and behavioural performance. Clinicians have a hard time, though, first of all because the role and influence of medical professionals is on the wane. Large groups of people self-confidently assert that they are very well capable of managing their own health. Commercial companies are all too happy to offer assistance: the market for web-based health monitoring tools, personal health advice, and healthy ageing coaches is booming. But even if people do seek medical advice, it is difficult to offer it in an evidence-based way. Deciding whether, when, and how to intervene becomes much more complex, since scientific research is hardly able to keep up with the data produced by ubiquitous and permanent monitoring. Although, as some reflexive clinicians note, this might be more like an illusion lost than a real change. With the benefit of hindsight, they state, past medical interventions now can be seen for what they always were: experiments lacking a sound scientific basis.

## 5. Deliberating Sociocultural Impacts

As said above, scenarios like the three presented here can be used to broaden the imagination as well as the deliberation of those who are involved or may be involved in the future. The scenarios also enable discussion of what is actually problematic in the current situation: what are the problems and needs surrounding AD and how might the attempt to detect AD early affect those problems and needs? Such exercises can be organised with stakeholders in various ways [31, 32]. Generally defined, stakeholders are (groups of) actors who are invested in a particular outcome of the envisioned technological developments, that is, they have something to gain or to lose from it [33, 34]. This is no doubt the case for patients (in particular future ones), clinicians (physicians, nurses, and all kinds of paramedics) involved in the diagnosis and care for AD patients, family members and other informal care givers, the technology developers (scientists, industry), clinical guideline developers, and insurance companies. But as the scenarios imply, impacts may be felt as well by the elderly in general, those caring for the elderly, employers, policy makers, and in the Dutch case even the committees assessing euthanasia cases. When inviting stakeholders for engagement activities, choices will have to be made; a careful balancing of the number of participants and their respective backgrounds is necessary to ensure an even handed deliberation process. What is feasible depends, of course, on the design of the deliberation (discussed below). In addition to the question which groups to include, the question how to find representatives of the groups to be included deserves attention. After all, not all individuals may be representative of the group they are supposed to speak for. In the case of AD, in particular, the inclusion of patients requires careful consideration. If it is feasible to find patients diagnosed with AD willing to participate, these will usually be at an early stage of the disease. To make sure that the voice of those familiar with later stages is also heard, inclusion of family members, nurses, or patient organizations should be considered.

Once stakeholders have been identified, the question is how to elicit their responses [32]. One method for doing this is to convene so-called “focus groups”: homogeneous groups of a particular type of stakeholders. In this case, several meetings with different groups are held, to probe the considerations of, say, patients and elderly, clinicians, and informal care givers, respectively. Having separate deliberations with these groups has several advantages. First, it enables the inclusion of a larger number of representatives of a specific group, thus possibly disclosing a variety of viewpoints among this group. In addition, it will be easier to discern to what extent specific considerations are widely shared within a specific group of stakeholders. Finally, in this setting, it will often be easier to create an atmosphere of trust, stimulating participants to voice all their considerations without worry about being overwhelmed by others. This may hold particularly for patients and caregivers. In general, then, focus groups enable the disclosure of a rich variety of considerations and viewpoints. Moreover, they lend themselves very well to a phased setup, in which first the problems with, as well as the good qualities of the present situation can be explored, before responses to ongoing developments are probed by presenting scenarios like the ones outlined above. The ultimate goal of any focus group is to list the conditions future developments should satisfy for a specific group of stakeholders to accept these developments. The conditions identified can be technological, social, organizational, financial, or cultural. As a result, who will be the addressees of the focus group's conclusion is an open question.

Another method for probing stakeholders' responses, which may be combined with the focus group method, is to bring different stakeholders together in a larger interactive setting. Since different groups of stakeholders may radically differ from, or even contradict, each other, it can be difficult to weigh or prioritize outcomes of separate focus groups. Moreover, it is unclear who is in the best position to perform this weighing. In addition, the moral legitimacy of all stakeholders' considerations can increase when these are checked and judged to be legitimate by a larger set of stakeholders. In an interactive, multistakeholder setting, chances will increase that the ultimate conclusions not just seek to further the interests of a specific group, but are based on widely shared values. Interactive deliberation enables the production of truly ethical judgments. Finally, the opportunities for creative thinking on how to address the legitimate concerns of specific stakeholders may increase, thus contributing not only to a well-considered problem analysis, but also to possible solutions or improvements. The actual process of deliberating the different scenarios will not differ very much from the one used in focus groups, although in an interactive setting the person chairing the session will have to be even more diligent in explicitly connecting participants to further listening and a serious consideration of all that is brought to the fore.

If feasible, then, having several focus groups first before bringing stakeholders together in an interactive workshop might combine the best of both worlds. In addition, a thorough exploration of stakeholders' views on the present situation of AD diagnostics (in a descriptive and an evaluative way) will help to keep the discussion focused on what needs to be done here and now, as well as to improve the understanding of participants' responses to the scenarios in the subsequent phase. By explicitly returning to the diagnosis of the present after having deliberated the imagined futures, these deliberations are most likely to result in conclusions as to what needs to be done, by whom, here and now to ensure responsible innovation.

## 6. Conclusion

In view of the current investments pursuing molecular technologies for early diagnosis of Alzheimer's disease, as well as the recent proposals for expanding diagnostic categories in guidelines for diagnosing this disease, it is important to evaluate the broader impacts these developments may have (and to some extent already have) on society and culture. We have argued that such an assessment might enhance the democratic character of technology development and lead to more robust technologies. We also indicated that, to be effective, such an assessment should take place at a relatively early stage of technology development. This paper outlines how one might proceed in three steps, focusing in particular on impacts on the way society views and deals with ageing. First, a conceptual analysis of the history of AD and aging provides valuable insights in the broader impact of AD diagnosis. Secondly, these insights can be used to create imaginative, plausible scenarios about the future, which might then, thirdly, be used as input for deliberation among stakeholders via focus groups and broad interactive workshops. Ultimately, such a process will help to identify the conditions for responsible innovation of AD diagnostics, by enabling innovators and societal stakeholders to become mutually responsive to each other's needs and values.
